# Insomnia management; don't sleep on it

**DOI:** 10.1192/bjo.2021.110

**Published:** 2021-06-18

**Authors:** Maria Donnelly, Nieves Mercadillo, Stuart Davidson

**Affiliations:** 1 Warrington and Halton Teaching hospitals; 2 North West Boroughs Healthcare NHS Foundation Trust

## Abstract

**Aims:**

In this project our aim was to improve patient safety and care by reducing hypnotic prescription medication administration. We also wanted to reduce over-prescribing/unnecessary prescribing which has a negative pharmaceutical impact on the environment and is a huge expenditure issue for the NHS. NICE guidance for Insomnia management states “After consideration of the use of non-pharmacological measures, hypnotic drug therapy is considered appropriate for the management of severe insomnia interfering with normal daily life; it is recommended that hypnotics should be prescribed for short periods of time only, in strict accordance with their licensed indications” Side effects are common with hypnotic usage including, most importantly, the development of tolerance and rebound insomnia.

**Method:**

The interventions we implemented included the development of an educational presentation about insomnia, the development of an “Insomnia Management Flow chart” to be used at admission point, training sessions for ward staff, shared teaching programmes with patients at their sleep management sessions, face to face and email correspondence to inform medical trainees about this project and gathering feedback from patients and staff before and after this project.

**Result:**

The results of this project demonstrated a total reduction in hypnotic tablet administration was very significant with a 44.5% reduction post intervention.

**Conclusion:**

This demonstrates the positive change in our clinical practice that has resulted from our interventions. This will improve patient safety and reduce cost of hypnotic medications for the NHS. Following on from this initial intervention, we feel that we can continue to make further changes and expand the changes we made on this ward, to other similar wards in our hospital, trust and to other inpatient psychiatric wards further afield.

